#  Correlation of Histomorphological Findings with Bacteriological Index in Leprosy Patients

**DOI:** 10.30699/IJP.2021.534122.2682

**Published:** 2021-12-15

**Authors:** Shramika Mahadev Naik, Swapnil Arun More, Sneha Ramdas Joshi

**Affiliations:** 1 Department of Pathology, M.I.M.E.R. Medical College, Talegaon (D), Pune, Maharashtra, India

**Keywords:** Bacteriological index, Hansen’s disease, Leprosy, Slit-skin smear

## Abstract

**Background & Objective:**

Leprosy is characterized by various clinicopathological forms depending on the host's body. Therefore, the correlation of histopathological findings with bacteriological index is helpful in diagnosing, classification, and monitoring the treatment. We aimed to analyze the histomorphological findings correlation with the bacteriological index in different types of leprosy. Then, study the histopathological spectrum of leprosy.

**Methods:**

We carried out a histomorphological study of skin biopsies obtained from 100 new patients tested clinically in OPD (Out Patients Department) on the basis and calculation of bacteriological index on a slit-skin smear. The histomorphological findings correlation with the bacteriological index was to be found in different types of leprosy.

**Results:**

In the histopathological studies, 52% of the patients were diagnosed with borderline tuberculoid (BT) followed by 20% with borderline lepromatous (BL), 13% with lepromatous leprosy (LL), 8% with tuberculoid (TT), 4% with histoid Hansen's disease, and 3% with mid-borderline (BB). On the clinical and histopathological examinations, correlation was found for 80% of the cases. Considering the correlation of histopathological features with the bacteriological index, 63% of the cases showed good correlation which was comparable with that of other studies.

**Conclusion:**

Because of the underlying symptoms of leprosy, there is a difference between different types of leprosy and the clinical and environmental perceptions. Thus, the correlation of clinical, histopathological, and bacteriological indices could be more helpful in the diagnosis of leprosy rather than considering only one parameter.

## Introduction

Leprosy is recognized as a granulomatous disease caused by Mycobacterium leprae in which skin is mainly affected. The pathogenesis of leprosy is complex and its clinicopathological manifestations are the result of host-parasite interactions (1, 2).

Although the prevalence is declining, the disease continues to be the major cause of many public health problems. It was found that 211903 new cases of leprosy were diagnosed in 2010, globally (3). The worst affected countries were India and Brazil as well as other countries in Sub-Saharan Africa and Southeast Asia (4). The mechanism of transmission is unknown; however, it is believed to be done through the inhalation of bacilli extracted from the compressed lungs of a multibacillary patient (5).

The disease manifests itself in two polar forms, namely lepromatous and tuberculoid leprosy, lying on both sides of a wide range. Between these two polar forms lie the borderline and intermediate forms (6).

The clinical presentation can range from a minor skin lesion to a serious condition where damage to the nerves, eyes, and bones can occur (5). The diagnosis of any type of leprosy in any patient depends on the body's response. Paucibacillary (tuberculoid end of spectrum) is the result of a strong cellular response (6).

In 1966, Ridley and Jopling proposed the leprosy classification as follows: tuberculoid (TT), borderline tuberculoid (BT), mid-borderline (BB), borderline lepromatous (BL), and lepromatous (LL). 

Bacteriological Index (BI) (7): The concentration of bacilli in smears is known as the bacterial or bacteriological index and includes living and dead bacilli. 

The most common index is Ridley's logarithmic measurement, which is based on the number of bacilli for the purpose of oil immersion.

- 6+ more than 1000 bacilli in an average field
- 5+ 100 to 1000 bacilli in an average field
- 4+ 10 to 100 bacilli in an average field
- 3+ 1 to 10 bacilli in an average field
- 2+ 1 to 10 bacilli in 10 fields
- 1+ 1 to10 bacilli in 100 fields

At least 100 immersion oil smears should be checked before reporting bacterial index slides.

**Slit-Skin Smear Examination**

 In 1935, Wade described a slit-skin smear method which was modified in 1947 (8). Slit-skin smear is a simpler and more important test compared to other leprosy diagnostic tests.

Role of slit-skin smear test: 1) To confirm the diagnosis, 2) To distinguish between the types, 3) To determine the effectiveness of the treatment, 4) To assess the progression of the disease, and 5) To follow-up.

Initially, smears are taken from many sites of the patients’ bodies, including the suspicious sites. According to recent studies, the number of sites has now been reduced to four due to the risk of HIV transmission (9). Currently, the four most common sites for biopsy are 1) lobe of the right ear, 2) forehead, 3) chin, and 4) left the gluteal region in the men and left upper thigh in the women.

Although many cases of leprosy can be diagnosed clinically without any histopathological examination, it is still considered an important test to reach a valid diagnosis. Therefore, the integration of clinical findings with histopathological ones is very important in disease management. A direct typing of leprosy is sometimes not possible in a clinic. Moreover, the side effects of skin rashes lead to a misdiagnosis. To prevent this, a histopathological examination should be performed in all suspected cases.

Early detection and on-time treatment may reduce the damage caused by the disease and make the person noninfectious.

Therefore, correlating the histomorphological findings with the bacteriological index obtained by skin smears could be helpful in diagnosing, isolating, and successful monitoring the treatment.

This study aimed to analyze the correlation of histomorphological findings with the bacteriological index in different types of leprosy, and to inspect the histopathological spectrum of leprosy.

## Material and Methods

**Study Design**

This study was a cross-sectional study that was done over a period of two years. Moreover, this study was conducted at the Tertiary Care Facility in the Department of Pathology.

A total of 100 patients who were clinically suspected of or diagnosed with leprosy prior to the beginning of MDT (Multi-drug therapy) and fulfilled the inclusion procedure were enrolled in this study.

Considering the 95% confidence level and the confidence interval of 10, the number of patients to achieve a statistical significance in our study was determined to be 96. This calculation is made by Survey System (http://www.surveysystem.com/sscalc.htm#one). The Survey System ignores the size of the population if it is "large" or unknown. The population size may only be a factor when working with a small known group of people (e.g., members of an organization).

**Inclusion And Exclusion Criteria**

Patients clinically suspected of or diagnosed with leprosy prior to the onset of MDT were included. Patients who were treated for leprosy were excluded from the study.

Methods: After approval by the Ethics Committee, the study began with informed legal consent. Once patients enrolled for the study, a complete history and physical examination were performed after obtaining the informed written consent.

The study material consisted of skin biopsies from multiple sites of the patients’ bodies who were clinically diagnosed with leprosy, as well as slit-skin smears from all the patients suspected of being diagnosed with leprosy, prior to the onset of MDT.

Biopsies (placed in 10% formalin) were sent to the Department of Pathology. Tissue sections were stained with hematoxylin and eosin (H&E) and Ziehl-Neelsen (ZN) (5%) to show the lepra bacilli.

The slit-skin smears were sent to the Department of Microbiology. 

The number and site of smear was determined according to the World Health Organization (WHO) recommendation for sampling.

In the ZN-stained smears, the total amount of bacilli was measured using the Ridley's logarithmic and bacteriological index.

After studying the histopathological features and noting the bacteriological status, the diagnosis of leprosy was confirmed, and the classification was done according to the Ridley-Jopling classification for leprosy, and the histomorphological correlation was made with the bacteriological index.

**Statistical Analysis**

Data were presented using the mean and standard deviations. Comparisons between the study groups were made using the unpaired t test as per results of normality tests. Moreover, the qualitative data were presented using the frequency and percentage. Interactions between the study groups were assessed by the Fisher’s exact test, student’s t test, and Chi-square test. A P-value less than 0.05 was considered as statistically significant.

Pearson's Chi-square test was calculated as follows:

$X^{2}=\sum_{i=1}^{n}\frac{{\left(O_{i}-E_{i}\right)}^{2}}{E_{i}}$

Where Χ2 is Pearson's cumulative test statistic, Oi is an observed frequency, Ei is an expected frequency, asserted by the null hypothesis, and n is the number of cells in the table**.**

## Results

Regarding the age, the majority of patients (35%) were in the 21-30 age group, followed by 21% in the 31-40 age group.

Considering the sex-wise distribution, the majority of patients (69%) were male, while the female patients accounted for 31% of the study population.

In terms of the primary site of lesion, the most common primary site was the upper extremities (35%), followed by the face (30%).

When it came to the bacteriological index (Ridley scale) in the patients (Table 1), it was 1+ and 2+ in 22% and 13% of the patients, respectively. In comparison, it was 3+ and 4+ in 5% and 10% of the patients, respectively, regarding (Ridley Scale). Moreover, 37% of the patients showed negative findings.

**Table 1:** Distribution of Patients According to Bacteriological Index (Ridley Scale)

Bacteriological Index	No.	%
0	37	**37%**
1+	22	**22%**
2+	13	**13%**
3+	5	**5%**
4+	10	**10%**
5+	8	**8%**
6+	5	**5%**
Total	100	**100%**

No.: Number of patients; %: Percentage

**Fig. 1:**
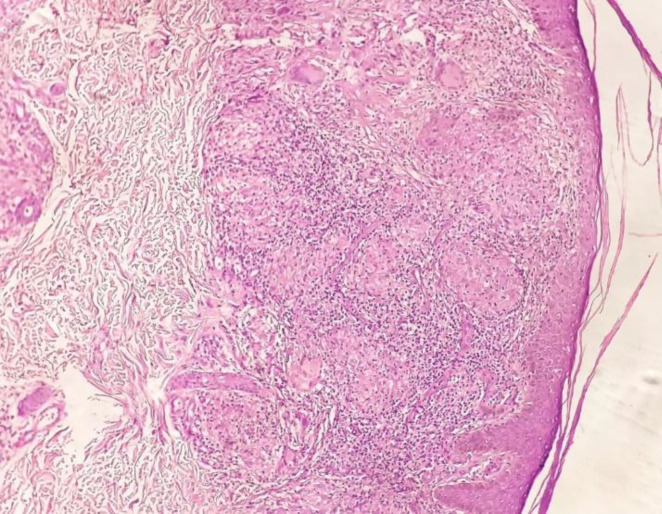
Photomicrograph of Tuberculoid Leprosy Showing Epithelioid Cell Granulomas with Langhan’s Giant Cells and Lymphocytes (H&E, 10x)

Furthermore, the most common histopathological diagnoses (Table 2) were BT (52%) followed by BL (20%), LL (13%), TT (8%), histoid hansen’s disease (4%), and BB (3%). Figure 1 shows the microscopy study of tuberculoid leprosy.

**Table 2:** Distribution of Patients According to Histopathological Diagnosis

Histopathological Diagnosis	No.	%
Borderline Tuberculoid (BT)	52	**52%**
Borderline Lepromatous (BL)	20	**20%**
Lepromatous Leprosy (LL)	13	**13%**
Tuberculoid (TT)	8	**8%**
Histoid Hansen’s Disease	4	**4%**
Mid-borderline (BB)	3	**3%**
Total	100	**100%**

No.: Number of patients; %: Percentage

As shown in Table 3, the correlation of histopatho-logical diagnosis and bacteriological index was seen in 63% of the cases. The highest correlation was seen in BL (100%), LL (100%), histoid hansen’s disease (100%), and BB (100%) followed by BT (44.2%) and TT (0%).

**Table 3:** Correlation of Histopathological Diagnosis and Bacteriological Index

HPE	0		1+		2+		3+		4+		5+		6+		Total	Correlation
	No	%	No	%	No	%	No	%	No	%	No	%	No	%		
BT	29	29%	21	21%	2	2%	0	-	0	-	0	-	0	-	52	**63%**
BL	0	-	0	-	9	9%	5	5%	4	4%	1	1%	1	1%	20	
LL	0	-	0	-	0	-	0	-	6	6%	4	4%	3	3%	13	
TT	8	8%	0	-	0	-	0	-	0	-	0	-	0	-	8	
Histoid Hansen’s Disease	0	-	0	-	0	-	0	-	0	-	3	3%	1	1%	4	
BB	0	-	1	1%	2	2%	0	-	0	-	0	-	0	-	3	
Total	37	37%	22	22%	13	13%	5	5%	10	10%	8	8%	5	5%	100	

No: Number of patients; %: Percentage; HE: Histopathological examination; BT: Borderline Tuberculoid; BL: Borderline Lepromatous; LL: Lepromatous Leprosy; TT: Tuberculoid; BB: Mid-borderline

According to Table 4, the maximum correlation of histopathological diagnosis with the clinical diagnosis was seen in BT (88.4%) followed by LL (77%), BL (75%), histoid Hansen's disease (75%), BB (66.7%), and TT (50%). The overall correlation of the histopathological diagnosis with the clinical diagnosis was 80%, which was a statistically significant correlation (*P*<0.05).

## Discussion

**Age-Wise Distribution**

In the current study, the majority of patients (35%) were in the 21-30 age group followed by 21% in the 31-40 age group, 16% in the 41-50, 9% in the 51-60, 7% in the 61-70, 5% in the 1-10 and 11-20, and 2% in the 71-80 age group. The mean age of the patients was 36.50 ± 15.52. These findings are comparable with those of Mehta* et al. *(9), Singh* et al. *(10), Namrata* et al. *(11), Baddam* et al. *(12), and Susmitha* et al. *(13). These authors found that the most common age group affected was 21-30 years of age followed by 31-40 age group.

**Sex Wise Distribution**

In the current study, the majority of patients (69%) were male while the female patients accounted for 31% of the study population. This finding corroborated that of the studies conducted by Singh* et al. *(10), Thamilselvi* et al. *(14), Kakkad* et al. *(15), Baddam* et al. *(12), and Susmitha* et al. *(13) who found that men were more commonly affected compared to the women. The men's prominence can be due to many consolidating factors (16).

**Primary Site of Lesions in Leprosy**

The most common primary sites of the lesion in the present study were upper extremities (35%) followed by face (30%), trunk (15%), lower extremities (12%), 

**Table 4:** Correlation of Histopathological Diagnosis with Clinical Diagnosis

HPE	BT		LL		BL		TT		BB		Histoid Hansen’s Disease		Neural Hansen		Total	Correlation
	No	%	No	%	No	%	No	%	No	%	No	%	No	%		
BT	46	46%	2	2%	0	-	3	3%	1	1%	0	-	0	-	52	**88.4%**
BL	0	-	3	3%	15	15%	1	1%	1	1%	0	-	0	-	20	**75%**
LL	2	2%	10	10%	0	-	0	-	0	-	0	-	1	1%	13	**77%**
TT	0	-	4	4%	0	-	4	4%	0	-	0	-	0	-	8	**50%**
Histoid Hansen	0	-	1	1%	0	-	0	-	0	-	3	3%	0	-	4	**75%**
BB	0	-	1	1%	0	-	0	-	2	2%	0	-	0	-	3	**66.7%**
Total	48	48%	21	21%	15	15%	8	8%	4	4%	3	3%	1	1%	100	

No: Number of patients; %: Percentage; HE: Histopathological examination; BT: Borderline Tuberculoid; BL: Borderline Lepromatous; LL: Lepromatous Leprosy; TT: Tuberculoid; BB: Mid-borderline.

head and neck (6%), and back (2%). This result is comparable with the findings of Tekwani* et al. *(17) and Shrestha* et al. *(18).

Tekwani* et al. *(17) studied the clinico-histopathological correlation in different types of leprosy and observed that the upper extremity was the primary site of lesion in 47 cases (34.81%), followed by the face in 40 cases (29.62%), trunk in 20 cases (14.81%), the lower extremities in 16 cases (11.85%), head and neck in 8 cases (5.92%), and back in 4 cases (2.96%).

In the descriptive study done by Shrestha* et al. *(18), it was found that the most common lesions were seen in the upper extremities of 15 cases (30%) followed by the lesions in all the body of 13 cases (26%). 

A study by Shubangi* et al. *(19) showed that the most common lesions were seen in the upper extremities comprising 37.8% of the cases, followed by back (30.2%) and the lower extremities (2%).

**Bacteriological Index**

In the present study, the bacteriological index was 0 in 37% of the cases.

This result lends support to the results of Rahul* et al. *(80%) (20), Tiwari* et al. *(22.6%) (21), Giridhar* et al. *(43.9%) (22), and Kakkad *et al.* (30%) (15).

It was observed in a recent study that the bacteriological index (Ridley scale) was 1+ and 2+ in 22 (22%) and 13 cases (13%), respectively. The bacteriological index in the paucibacillary patients was also seen in the studies of Susmitha* et al. *(21.6%) (23), Tiwari* et al. *(33.8%) (21), and Kakkad* et al. *(50%) (15).

The bacteriological index was 3+ and 4+ in 5 (5%) and 10 patients (10%), respectively, and 5+ and 6+ in 8 (8%) and 5 patients (5%), respectively. The multibacillary cases in the present study were 55 (55%). The bacteriological index in the multibacillary patients was comparable with that index in the studies of Tiwari* et al. *(66.2%) (21), Giridhar* et al. *(24.5%) (22), and Kakkad* et al. *(50%) (15). 

**Histopathological Diagnosis of Leprosy**

In the present study, the most common histopathological diagnosis was related to the BT patients (52%). Similar observations were noted in the studies of Tekwani* et al. *(57.77%) (17), Nadia* et al. *(34.7%) (24), Kadam* et al. *(35.7%) (25), Singh* et al. *(31.7%)) (10), and Mehta* et al. *(26%) (9).

The second most common histopathological diagnosis in the current study was related to the patients with LL (20%). Similar observations were noted in the studies of Nadia* et al. *(21.2%) (24), Singh* et al. *(13.3%) (10), and Mehta* et al. *(20%) (9), while only 5.18% of the cases and 9.5% of the cases were identified in the studies conducted by Tekwani* et al. *(17) and Kadam* et al. *(25), respectively.

BL was detected in 13 cases (13%) in the present study. This result is in line with the results achieved by Tekwani* et al. *(14.81%) (17), Singh* et al. *(21.7%) (10), and Mehta* et al. *(25%) (9). Only 5.9% and 4.8% of the cases were identified in the studies conducted by Nadia* et al. *(24) and Kadam* et al. *(25), respectively.

TT was detected in 8 cases (8%) in the present study. This is consistent with the studies of Nadia* et al. *(14.4%) [24] and Singh* et al. *(10%) (10) while most TT cases were found in the studies of Tekwani* et al. *(19.25%) [18], Kadam* et al. *(19%) (25), and Mehta* et al. *(26%) (9).

Furthermore, BB was detected in 3 cases (3%) in the present study. This result is consistent with the results of Tekwani* et al. *(0.74%) (17), Kadam* et al. *(2.4%) (25), and Mehta* et al. *(3%) (9). The majority of BB cases were found in the studies conducted by Nadia* et al. *(16.1%) (24) and Singh* et al. *(13.3%) (10).

Histoid leprosy was detected in 4% of the cases in the present study. This result is similar to that of the studies conducted by Tekwani* et al. *(2.22%) (17), Nadia* et al. *(3.4%) (24), Kadam* et al. *(4.8%) (25), and Singh* et al. *(4.2%) (10). No case of histoid leprosy was found in the study of Mehta* et al. *(9).

**Correlation of Histopathological Diagnosis and Bacteriological Index**

In the current study, there was a 63% correlation between the histopathological diagnosis and the bacteriological index. The highest correlation was seen in the BL (100%), LL (100%), histoid hansen’s disease (100%), and BB (100%), followed by BT (44.2%) and TT patients (0%). This finding is in line with the results of Premalatha* et al. *(26), Tekwani D* et al. *(16), Tiwari* et al. *(21), Pashupathy* et al. *(27), Giridhar* et al. *(22), and Murugnantham* et al. *(28).

Premalatha* et al. *(26) classified the leprosy into various types according to the bacillary index, morphological findings both in the slit-skin smears, and biopsy along with the clinical correlation. The association between the slit-skin smears and histopathological diagnosis showed that TT, BT, and BB strains did not fit well and the percentage of diagnoses was lower than that of TT (0%), BT (66.6%), and BB types (62.5%). In the BL and HL models, the diagnosis made in the slit-skin smears was 100% consistent with the histopathological diagnosis and only in the LL type, the slit-skin smears was 88.8% consistent with the histopathological diagnosis.

Giridhar* et al. *(22) showed the highest correlation between the histopathological diagnosis and the slit-skin smear testing in the BL (100%), LL (100%), and TT (100%) types. The least correlation was observed in the BT patients (30.95%).

Tekwani* et al. *(17) reported the majority of patients as paucibacillary patients (69.72%) and the rest were multibacillary ones (30.37%). All the BL and LL cases had multibacillary leprosy.

Tiwari* et al. *(21) showed the slit-skin smear positivity in 55% of the cases. The bacillary index was <2 in the TT and > 2 in the LL type.

In the BT type, the bacteriological index ranged from 0 to 2+, in BL 3+ to 6+, in LL 5+ to 6+, and in TT 0 to 1+.

Mridula* et al. *(13) showed the Acid Fast Bacilli (AFB) positivity in various types of leprosy. All the TT cases were AFB negative. LL showed 66.7% AFB positive cases followed by BB (33.3%), BT (28.6%), and BL (25%) cases.

**Clinico-Histopathological Correlation**

In the present study, the overall clinico-histopatho-logical correlation was 80%, which is consistent with that of the studies done by Tekwani* et al. *(72.59%) (17), Kakkad* et al. *(84%) (15), Singh* et al. *(81.6%) (10), and Moorthy* et al. *(62.6%) (29).

In our study, maximum correlation was seen in the BT type (88.4%), corroborating the studies done by Tekwani* et al. *(79.96%) (17), Kakkad* et al. *(83.33%) (15), and Singh* et al. *(83.3%) (10). Murugnantham* et al. *(28) found correlation only in 25% of the BT cases.

The second highest correlation was observed in the LL patients (77%), comparable to the studies conducted by Tekwani* et al. *(85.7%) (17), Kakkad* et al. *(93.3%) (15), Singh* et al. *(70%) (10), and Moorthy* et al. *(80%) (29). Tekwani* et al. *(17) found a correlation of only 50% in the LL patients. Regarding the BL type, correlation was seen in 75% of the cases in the present study. This is in line with the studies done by Tekwani* et al. *(54.16%) (17), Kakkad* et al. *(60%) (15), and Moorthy* et al. *(70%) (29). Maximum correlation in the BL type was seen by Singh* et al. *(94.7%) (10).

The correlation of BB was seen in 66.7% of the cases in the present study. This is similar to the studies conducted by Murugnantham* et al. *(50%) (28), Kakkad* et al. *(50%) (15), Singh* et al. *(75%) (10), and Moorthy* et al. *(50%) (29). Tekwani* et al. *(17) found only 25% correlation.

The incidence of TB leprosy was detected in only 50% of the cases in the present study. This result is similar to that obtained in the studies conducted by Murugnantham* et al. *(57.1%) (28) and Moorthy* et al. *(46.15%) (29). Kakkad* et al. *(15) and Singh* et al. *(10) found a 100% correlation in the TT patients. Tekwani D* et al. *(17) found a correlation of 83.33%.

Clinico-histopathological correlation in the histoid leprosy was observed in 75% of the cases in the present study. Histoid leprosy showed a 100% correlation in the studies conducted by Murugnantham* et al. *(28), Semwal* et al. *(30), and Tekwani D* et al. *(17), as well as 71.4% in the study conducted by Singh* et al. *(10).

**Summary**

In the current study, following observations were made:

1. Most patients (35%) were in the 21-30 age group followed by 21% in the 31-40 age group, 16% in the 41-50 age group, 9% in the 51-60 age group, 7% in the 61-70 age group, 5% in the 1-10 and 11-20 age groups, and 2% in the 71-80 age group. The mean age of the patients was 36.50 ± 15.52.

2. The majority of patients (69%) were male, while the female patients accounted for 31% of the study population.

3. The most common primary site of the lesion was upper extremities (35%) followed by face (30%), trunk (15%), lower edges (12%), head and neck (6%), and back (2%).

4. The bacteriological index (Ridley scale) was 1+ and 2+ in 22 (22%) and 13 patients (13%), respectively, while 3+ and 4+ in 5 (5%) and 10 patients (10%), respectively. Furthermore, the bacteriological index was 5+ and 6+ in 8 (8%) and 5 patients (5%), respectively. This is while the bacteriological index was 0 in 37 patients (37%).

5. The most common histopathological diagnoses were related to borderline tuberculoid (BT) (52%) followed by borderline lepromatous (BL) (20%), lepromatous leprosy (LL) (13%), tuberculoid (TT) (8%), histoid –hansen’s disease (4%), and mid-borderline (BB) (3%).

6. The overall correlation of the histopathological diagnosis with the bacteriological index was 63%. The highest correlation of histopatho-logical diagnosis and the bacteriological index was seen in the LL (100%), BL (100%), histoid (100%), BB (100%), and BT types (44.2%).

Complete improvement of the histopathological diagnosis and clinical diagnosis was seen in the BT (88.4%) followed by LL (77%), BL (75%), histoid leprosy (75%), BB (66.7%), and TT types (50%). The overall correlation of the histopathological diagnosis and clinical diagnosis was 80%, which was a statistically significant correlation (*P*<0.05). 

## Conclusion

The range of leprosy manifestations is very wide and there is a great variation between different types of leprosy; hence both clinical and histopathological factors and bacteriological indicators are more useful than any single parameter in achieving a definitive diagnosis and classification of the disease.

The histopathological examination should be performed in all cases for the proper diagnosis of leprosy; this may assist in better provision of the patients with the appropriate treatment.

The correlation of clinical features and histopatho-logical diagnosis with a bacteriological index seems to be more helpful in typing the leprosy than any of the individual parameters alone. This helps physicians to provide better patient care and management.

## Conflict of Interest

The authors declared no conflict of interest.

## Funding

None.
